# Supplementary material for: Pallidal neuromodulation of the explore/exploit trade-off in decision-making
Source: eLife. 2023 Feb 2;12:e79642. doi: 10.7554/eLife.79642 (PMC9940911; doi:10.7554/eLife.79642)
Supplement: Table 1—source data 1. — Estimated means (m) for ON and OFF-DBS conditions as well as contrast for ON-OFF. HDI values are for 95% highest density Interval. *Significant differences in the posterior distributions of the parameter estimates are highlighted in bold. [file elife-79642-table1-data1.docx]

| **Parameter** | **ON-DBS** | | | **OFF-DBS** | | | **Contrast** | | | |
| --- | --- | --- | --- | --- | --- | --- | --- | --- | --- | --- |
|  | ***mean*** | **HDI** | | ***mean*** | **HDI** | | ***mean*** | **HDI** | |  |
| *Pre-reversal* |  |  |  |  |  |  |  |  |  |  |
| Boundary Separation | 1.94 | 1.63 | 2.27 | 2.07 | 1.76 | 2.50 | -0.18 | -0.62 | 0.26 |  |
| Drift rate Scaling | 4.28 | 3.07 | 5.72 | 5.00 | 3.70 | 6.66 | -0.73 | -2.78 | 1.10 |  |
| Learning rate + | -0.46 | -1.70 | 1.05 | -0.82 | -1.99 | 0.51 | 0.33 | -1.36 | 2.29 |  |
| Learning rate - | -2.96 | -5.11 | -1.67 | -4.00 | -6.71 | -2.48 | 1.15 | -1.40 | 3.79 |  |
| *Post-reversal* |  |  |  |  |  |  |  |  |  |  |
| Boundary Separation | 1.66 | 1.48 | 1.90 | 1.83 | 1.60 | 2.10 | -0.15 | -0.47 | 0.15 |  |
| Drift rate Scaling | 1.5 | 0.55 | 2.63 | 3.4 | 2.03 | 4.69 | **-1.71*** | **-3.4** | **-0.18** |  |
| Learning rate + | 2.16 | 0.80 | 6.78 | 0.34 | -1.96 | 3.51 | 2.21 | -2.57 | 6.81 |  |
| Learning rate - | -0.76 | -3.34 | 1.88 | -3.09 | -5.81 | -0.99 | 2.02 | -1.14 | 6.07 |  |
