# Supplementary material for: Pallidal neuromodulation of the explore/exploit trade-off in decision-making
Source: eLife. 2023 Feb 2;12:e79642. doi: 10.7554/eLife.79642 (PMC9940911; doi:10.7554/eLife.79642)
Supplement: Table 1—source data 2. — Estimated means (m) for HC as well as contrast estimates for both DBS conditions HC-OFF and HC-ON. HDI values are for 95% highest density interval. *Significant differences in the posterior distributions of the parameter estimates are highlighted in bold. [file elife-79642-table1-data2.docx]

**Table 1 - Table Supplement - 2 Summary of Posterior distributions (RLDDM model fitted to whole task)**

| **Parameter** | **Healthy controls** | | | **Contrast (OFF-DBS)** | | | | **Contrast (ON-DBS)** |
| --- | --- | --- | --- | --- | --- | --- | --- | --- |
|  | ***mean*** | **HDI** | | ***mean*** | **HDI** | |  | ***mean* HDI** |
| Boundary Separation | 1.76 | 1.70 | 2.18 | -0.16 | -0.45 | 0.11 |  | -0.03 -0.26 0.25 |
| Drift rate Scaling | 2.76 | 2.23 | 3.31 | **-1.67*** | **-2.93** | **-0.71** |  | -0.81 -2.02 0.69 |
| Learning rate + | -0.63 | -1.81 | 0.88 | 0.22 | -0.91 | 1.39 |  | -0.99 -2.59 0.80 |
| Learning rate - | -1.68 | -2.25 | -0.95 | **1.32*** | **0.26** | **2.5** |  | 0.93 -0.05 2.12 |
