# Supplementary material for: Pallidal neuromodulation of the explore/exploit trade-off in decision-making
Source: eLife. 2023 Feb 2;12:e79642. doi: 10.7554/eLife.79642 (PMC9940911; doi:10.7554/eLife.79642)
Supplement: Supplementary file 1. — TWSTRS: Toronto Western Spasmodic Torticollis Rating Scale; BDI: Beck’s Depression Inventory; F: Female; M: Male; S.E.M: standard error of mean; L: left; R: right. *These patients only performed the task in one stimulation condition. [file elife-79642-supp1.docx]

| **Demographics** | | | | | **Stimulation details** | | **TWSTRS-Score** | | | | | | **Tsui-Score** | | | **BDI** | |
| --- | --- | --- | --- | --- | --- | --- | --- | --- | --- | --- | --- | --- | --- | --- | --- | --- | --- |
| **ID** | **Sex** | **Age** | **Disease Duration** | **DBS Duration** | **Parameters** | **Stim Start** | **Severity ON** | **Severity OFF** | **Impairment ON** | **Pain ON** | **Total ON** | **TSUI ON** | | **TSUI OFF** | **BDI** | |  |
| **0** | F | 54 | 22 | 8 | L: 10-, 3.1V; R: 1-, 3.1V; 130Hz, 90µs | OFF | n.a. | n.a. | n.a. | n.a. | n.a. | n.a. | | n.a. | n.a. | |  |
| **1** | F | 56 | 12 | 8 | L: 9-, 1.1V; R: 1-, 1.7V; 210Hz, 120µs | ON | 9 | 15 | 2 | 3.5 | 14.5 | 2 | | 8 | 2 | |  |
| **2** | F | 45 | 5 | 2 | L: 1-, 2-, 1.9V; R: 10-, 11-, 3.6V; 210Hz, 90µs | OFF | 3 | 6 | 1 | 9.25 | 13.25 | 1 | | n.a. | 17 | |  |
| **3** | F | 55 | 8 | 2 | L: 3-, 3mA; R: 11-, 3mA; 185Hz, 90µs | ON | 7 | 23 | 0 | 1.75 | 8.75 | 2 | | 10 | 6 | |  |
| **4** | M | 67 | 32 | 8 | L: 9-, 10-, 3.1V; R: 1-, 2-, 2.6V; 180Hz, 90µs | OFF | 6 | 7 | 17 | 11.75 | 34.75 | 2 | | 4 | 11 | |  |
| **5** | M | 55 | 21 | 10 | L: 9-, 2.2V; R: 1-, 4V; 60Hz, 90µs | OFF | 2 | 4 | 0 | 2 | 4 | 2 | | 2 | 4 | |  |
| **6** | F | 61 | 19 | 5 | L: K4- (30%), K5- (70%), 3.3mA; R: 12- (30%), K13- (70%); 231Hz, 120µs | ON | 14 | 15 | 13 | 15 | 42 | 7 | | 8 | 10 | |  |
| **7** | M | 56 | 17 | 14 | L: K9-, 2.1V; R: 1-, 2.1V; 130Hz, 90µs | ON* | 6 | n.a. | 8 | 0.75 | 14.75 | 2 | | n.a. | n.a. | |  |
| **8** | F | 61 | 14 | 10 | L: K9-, 10-, 1.5V; R: 1-, 2-, 1.8V; 180Hz, 90µs | OFF | 3 | 4 | 3 | 0 | 3 | 1 | | 2 | 1 | |  |
| **9** | F | 73 | 11 | 0 | L: 2-, 3-, 2.5mA; R: 10-, 11-, 2.5mA; 130Hz, 60µs | ON | 16 | 19 | n.a. | n.a. | n.a. | 9 | | 10 | 7 | |  |
| **10** | F | 63 | 32 | 1 | L: 2-, 3-, 2.9mA; R: 11-, 12-, 2.6mA, 130Hz, 90µs | ON* | 13 | 11 | 15 | 1 | 29 | 8 | | 8 | 21 | |  |
| **11** | F | 68 | 15 | 11 | L: 9-, 10-, 11- vs. 8+, 3.1V; R: 1-, 2-, 3- vs. 0+, 2.5V; 200Hz, 60µs | ON* | 6 | 21 | 10 | 7 | 23 | 3 | | 9 | 22 | |  |
| **12** | M | 56 | 22 | 11 | L: 10-, 11-, 2.8V; R: 2-, 3-, 2.1V; 60Hz, 120µs | OFF | 11 | 12 | 10 | 12.5 | 33.5 | 7 | | 7 | 16 | |  |
| **13** | F | 70 | 37 | 11 | L: 10-, 3.3V, 140Hz, 90µs; R: 1-, 2.8V, 140Hz, 120µs | ON* | 23 | n.a. | 17 | 14.25 | 54.25 | 10 | | n.a. | 10 | |  |
| **14** | M | 43 | 19 | 2 | L: 2-, 5.4mA; R: 2-, 3.3mA; 130Hz, 90µs | OFF | 15 | 18 | 6 | 4.75 | 25.75 | 10 | | 12 | 4 | |  |
| **15** | F | 54 | 29 | 18 | L: 5-, 6-, 3.0V; R: 1-, 2-, 3.7V; 180Hz, 90µs | ON* | 5 | n.a. | 5 | 0 | 10 | 3 | | n.a. | 20 | |  |
| **16** | F | 71 | 17 | 4 | L: 7-, 2.1V; R: 1-, 2.5 V; 65Hz, 60µs | ON | 4 | 8 | 2 | 6 | 12 | 2 | | 6 | 6 | |  |
| **17** | F | 71 | 26 | 6 | L: 9-, 3.1V; R: 0-, 3.2V; 140Hz, 90µs | OFF | 4 | 10 | 1 | 8 | 13 | 2 | | 3 | 9 | |  |
| **18** | F | 59 | 31 | 3 | L: 8-, 9-, 2.2V; R: 0-, 1-, 2V; 200Hz, 90µs | OFF | 3 | 5 | 0 | 2.5 | 5.5 | 0 | | 1 | 20 | |  |
| **Mean** |  | 59.79 | 20.47 | 7.13 |  |  | 8.33 | 11.87 | 6.47 | 5.88 | 20.06 | 4.06 | | 6.43 | 10.94 | |  |
| **S.E.M.** |  | 1.93 | 2.03 | 1.14 |  |  | 1.37 | 1.64 | 1.49 | 1.24 | 3.52 | 0.80 | | 0.93 | 1.71 | |  |
|  | | | | | | | | | | | | | | | | | |
