# Supplementary material for: Pallidal neuromodulation of the explore/exploit trade-off in decision-making
Source: eLife. 2023 Feb 2;12:e79642. doi: 10.7554/eLife.79642 (PMC9940911; doi:10.7554/eLife.79642)
Supplement: Supplementary file 2. — ACC, anterior cingulate cortex; BA, Brodmann area; CBM, cerebellum; IFG, inferior frontal gyrus, ins., insula; ITG, inferior temporal gyrus; MCC, midcingulate cortex; MedFG, medial frontal gyrus; MFG, middle frontal gyrus; MTG, middle temporal gyrus; OG, orbital gyrus; OL, occipital lobule; PCC, posterior cingulate cortex; PreCG, precentral gyrus; Prec, precuneus; PostCG, postcentral gyrus; SFG, superior frontal gyrus; SMG, supramarginal gurys; SNr, substantia nigra; STG, superior temporal gyrus. [file elife-79642-supp2.docx]

|  |  |  |  |
| --- | --- | --- | --- |

| *Positive Peak Coordinates X/Y/Z (Value)* | *Negative Peak Coordinates X/Y/Z (Value)* |  |
| --- | --- | --- |

| VLPFC (BA 45) | RH | 56/28/18 (0.58) | CBM | RH | 32/-36/-24 (-0.64) |
| --- | --- | --- | --- | --- | --- |
|  | LH | -56/22/12 (0.50) |  | LH | -30/-50/-20 (-0.60) |
| CBM | RH | 38/-82/-26 (0.49) | OL (BA 18) | RH | 36/-94/-12 (-0.62) |
|  | LH | -48/-62/-38 (0.65) |  | LH | -4/-98/-12 (-0.61) |
| Uncus (BA 36) | RH | 30/-4/-38 (0.48) | PostCG (BA 5) | RH | 26/-42/72 (-0.45) |
|  | LH | -22/4/-46 (0.41) |  | LH | -24/-36/64 (-0.37) |
| SFG (BA 8) | RH | 8/34/52 (0.60) | Prec (BA 7) | RH | 12/-46/64 (-0.60) |
|  | LH | -1/34/54 (0.57) |  | LH | -4/-74/38 (-0.59) |
| DLPFC (BA 9) | RH | 58/24/20 (0.59) | Midbrain | RH | 8/-32/-14 (-0.51) |
|  | LH | -56/22/20 (0.50) |  | LH | -8/-30/-12 (-0.49) |
| MTG (BA 21) | RH | 60/-24/-18 (0.58) |  |  |  |
|  | LH | -66/-52/-2 (0.54) |  |  |  |
| MedFG (BA 6, 9) | RH | 4/42/30 (0.54) |  | | |
|  | LH | -2/42/34 (0.53) |  |  |  |
| SMG (BA 40) | RH | 60/-48/36 (0.59) |  |  |  |
|  | LH | -54/-52/34 (0.48) |  |  |  |
| ITG (BA 21) | RH | 60/-10/-28 (0.54) |  |  |  |
|  | LH | -64/-18/-24 (0.51) |  |  |  |
| ACC (BA 32) | RH | 12/38/12 (0.54) |  |  |  |
|  | LH | -14/40/8 (0.43) |  |  |  |
| MCC (BA 24) | RH | 4/-4/30 (0.45) |  |  |  |
|  | LH | -1/-6/30 (0.46) |  |  |  |
| PreCG (BA 4) | RH | 60/-12/40 (0.52) |  |  |  |
|  | LH | -64/-12/30 (0.55) |  |  |  |
| PostCG (BA 1) | RH | 58/-14/44 (0.52) |  |  |  |
|  | LH | -60/-14/38 (0.53) |  |  |  |
| SMA (BA 6) | RH | 33/-10/65 (0.42) |  |  |  |
|  | LH | -31/-10/65 (0.36) |  |  |  |
| Putamen | RH | 32/-6/-10 (0.56) |  |  |  |
|  | LH | -24/2/-10 (0.47) |  |  |  |
